# Development of a Hydrogel Platform with GBM and Microglia: A Potential Glioblastoma Tumor Model

**DOI:** 10.1021/acsabm.5c00735

**Published:** 2025-08-25

**Authors:** Seyma Isik, Deniz Yucel, Vasif Hasirci

**Affiliations:** † Department of Medical Biotechnology, Graduate School of Health Sciences, 162328 Acibadem University, Atasehir, 34752 Istanbul Türkiye; ‡ Biomaterials Center, Acibadem University, Atasehir, 34752 Istanbul Türkiye; § Department of Histology and Embryology, Acibadem University, Atasehir, 34752 Istanbul Türkiye; ∥Departments of Biomaterials and ⊥Biomedical Engineering, Acibadem University, Atasehir, 34752 Istanbul Türkiye; # BIOMATEN, Center of Excellence in Biomaterials and Tissue Engineering, Middle East Technical University, Cankaya 06800 Ankara Türkiye

**Keywords:** Glioblastoma, brain decellularized ECM, hyaluronic acid methacrylate, *in vitro* model, tissue engineering

## Abstract

Glioblastoma (GBM) is an aggressive brain tumor with a complex microenvironment shaped by a dense extracellular matrix (ECM) and dynamic interactions with stromal cells, presenting major challenges for *in vitro* modeling. In this study, we developed a biomimetic hydrogel platform by integrating a brain-derived decellularized extracellular matrix (dECM) with hyaluronic acid methacrylate (HAMA), yielding a composite (1H3D) that closely reflects the ECM characteristics of GBM tissue. Mechanically, 1H3D hydrogels exhibited a compressive modulus of 9.44 ± 0.73 kPa and an elastic modulus of 458.30 ± 13.39 Pa, resembling native GBM tissue. By retaining biochemical components from the brain dECM, hydrogels support key cellular processes such as adhesion, matrix remodeling, and invasion. These functions are essential for mimicking the highly invasive, plastic, and adaptive behavior of glioblastoma, thereby enhancing the physiological relevance of the *in vitro* platform. Coculture with microglia promoted glioblastoma progression, as evidenced by a 43% increase in *K*_i_-67 expression and a 41% increase in invasion distance, underscoring the protumoral role of microglia–glioblastoma interactions within the engineered microenvironment. Altogether, integration of a GBM relevant hydrogel matrix with microglia coculture provides a biologically and mechanically representative *in vitro* platform that reproduces key features of tumor–stroma interactions, offering a useful tool for studying glioblastoma progression and enhancing the translational potential of preclinical models.

## Graphical abstract

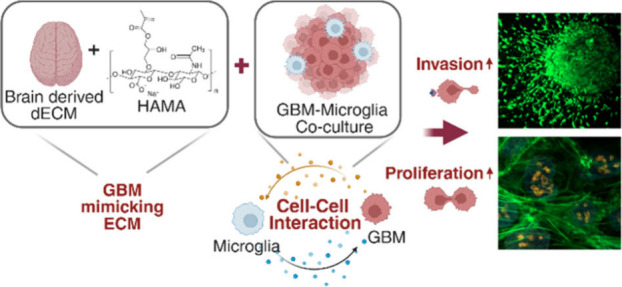

## Introduction

Glioblastoma (GBM) is a highly aggressive brain tumor with poor prognosis and high recurrence rates. GBM arises from genetic mutations and dysregulated growth factor pathways, which are resistant to current treatments.^1^ Despite standard therapy, surgical resection followed by radiotherapy (RT) and Temozolomide (TMZ) treatment, the median survival remains only 14.6 months, with a 2 year survival rate of only 26.5%.^2^ Despite advances in the understanding of GBM biology, its prognosis remains poor due to factors such as invasive growth, cellular heterogeneity, and a complex tumor microenvironment. The microenvironment plays a crucial role in promoting tumor progression, invasion, and resistance to therapy.^3^

Extracellular matrix (ECM) is a critical component in the GBM tumor microenvironment and profoundly influences tumor progression, invasion, and resistance to therapy. Unlike the healthy brain ECM, which is predominantly composed of hyaluronic acid (HA), glycosaminoglycans (GAGs), and proteoglycans with minimal fibrous proteins, GBM ECM undergoes extensive remodeling during cancer progression, altering both its composition and mechanical properties.^4^ In the healthy brain, HA constitutes a key component of the ECM, ensuring a hydrated, soft matrix critical for normal brain physiology.^5^ In GBM, HA levels increase significantly, facilitating the dynamic remodeling of the tumor microenvironment. Other ECM components also show marked upregulation in GBM.^4^ The biomechanical properties of the ECM in GBM are profoundly altered, contributing to tumor aggressiveness. Healthy brain ECM is soft and elastic, with a stiffness of approximately 1 kPa.^6,7^ In GBM, the stiffness is significantly higher, with compressive modulus values reaching up to 11.4 ± 4.9 kPa.^8^ Stiffened ECM activates mechanotransduction pathways via integrin signaling, promoting tumor cell migration, invasion, and survival. It also acts as a physical barrier that restricts immune cell infiltration and drug delivery, thus complicating the therapeutic efforts. These biomechanical differences not only support tumor progression but also present formidable challenges to treatment, highlighting the ECM as a critical target in GBM therapy.^9,10^

Recreating the molecular complexity and heterogeneity of ECM is crucial for improving the relevance of *in vitro* models. Although significant progress has been made in developing biomimetic ECMs using both natural and synthetic materials, fully replicating the intricate composition of native ECM remains a challenge. Traditional approaches often rely on simplified ECM compositions that fail to mimic the complexity of the native environment. In contrast, ECM derived from decellularized tissues better represents the native composition, offering a more biologically relevant microenvironment. Decellularized brain tissue has emerged as a key focus in regenerative medicine and tissue engineering. This process removes cellular components while maintaining the ECM’s biochemical properties, which are critical for cell interactions and functions. Several decellularization techniques exist, including physical, chemical, and enzymatic methods, however each has specific limitations. The effectiveness of decellularization is often assessed based on the residual DNA content and the retention of key ECM components, such as GAGs and proteins. These decellularized brain ECMs show potential for advancing *in vitro* models, offering a more accurate representation of the microenvironment.

This study aimed to develop a *in vitro* GBM model that closely replicates the native ECM of GBM, thereby providing a more accurate biological and mechanical platform for studying GBM biology and therapeutic responses. Previous models have used biological macromolecules, such as hyaluronic acid, collagen, fibrinogen, and decellularized tissues.^11–13^ Although these models mimic certain structural and compositional features of the tumor matrix, they still lack the full biochemical and mechanical complexity found in the native ECM. For example, hyaluronic acid–based models do not include essential ECM components, such as specific polysaccharides and structural proteins, which are critical for accurately representing the tumor microenvironment.^11^ Decellularized ECM derived from brain tissue retains the molecular and structural characteristics of brain ECM, enhancing the biological accuracy of the model.^14^ However, decellularized brain ECM alone has limitations, such as mechanical weakness and reduced hyaluronic acid content during the decellularization process. Additionally, applications with 3D bioprinting face challenges, such as low resolution and prolonged cross-linking times (approximately 1 h), which hinder the maintenance of structural integrity postprinting and the creation of complex structures.^15^

This study introduces a hydrogel platform that combine hyaluronic acid methacrylate (HAMA) and decellularized extracellular matrix (dECM) derived from brain tissue. By integrating the biochemical content of dECM with the tunable mechanical properties of HAMA, this platform not only mimics the native ECM more accurately, but also supports key tumor behaviors, such as invasion, adhesion, and matrix remodeling. Furthermore, microglia, as key stromal cells within the GBM microenvironment, were cocultured with glioblastoma cells, resulting in a pronounced enhancement of the proliferative and invasive capacities of glioblastoma cells. This observation underscores the pivotal role of microglia in shaping the tumor microenvironment and actively facilitating tumor progression and invasion through their interactions with glioblastoma cells.

## Results and Discussion

### Assessment of Decellularization Efficiency of the Brain Tissue

Physical decellularization using the freeze–thaw cycle method in combination with treatment with the nonionic detergent Triton X-100 was employed to remove cellular components from the brain tissue (dECM 1). For comparison of an efficient cleansing, an alternative decellularization protocol (dECM 2) utilizing sodium dodecyl sulfate (SDS) involving DNase was also tested. The dECMs obtained from these protocols were analyzed to determine their decellularization efficiency. The removal of cellular components was assessed using 4′,6-diamidino-2-phenylindole (DAPI) and Hematoxylin & Eosin (H&E) staining. The results demonstrated the efficiency of the two protocols, with the removal of nearly all cell nuclei from the native brain tissue, while preserving the structural integrity of the extracellular matrix (Figure 1A).

**Figure 1:**
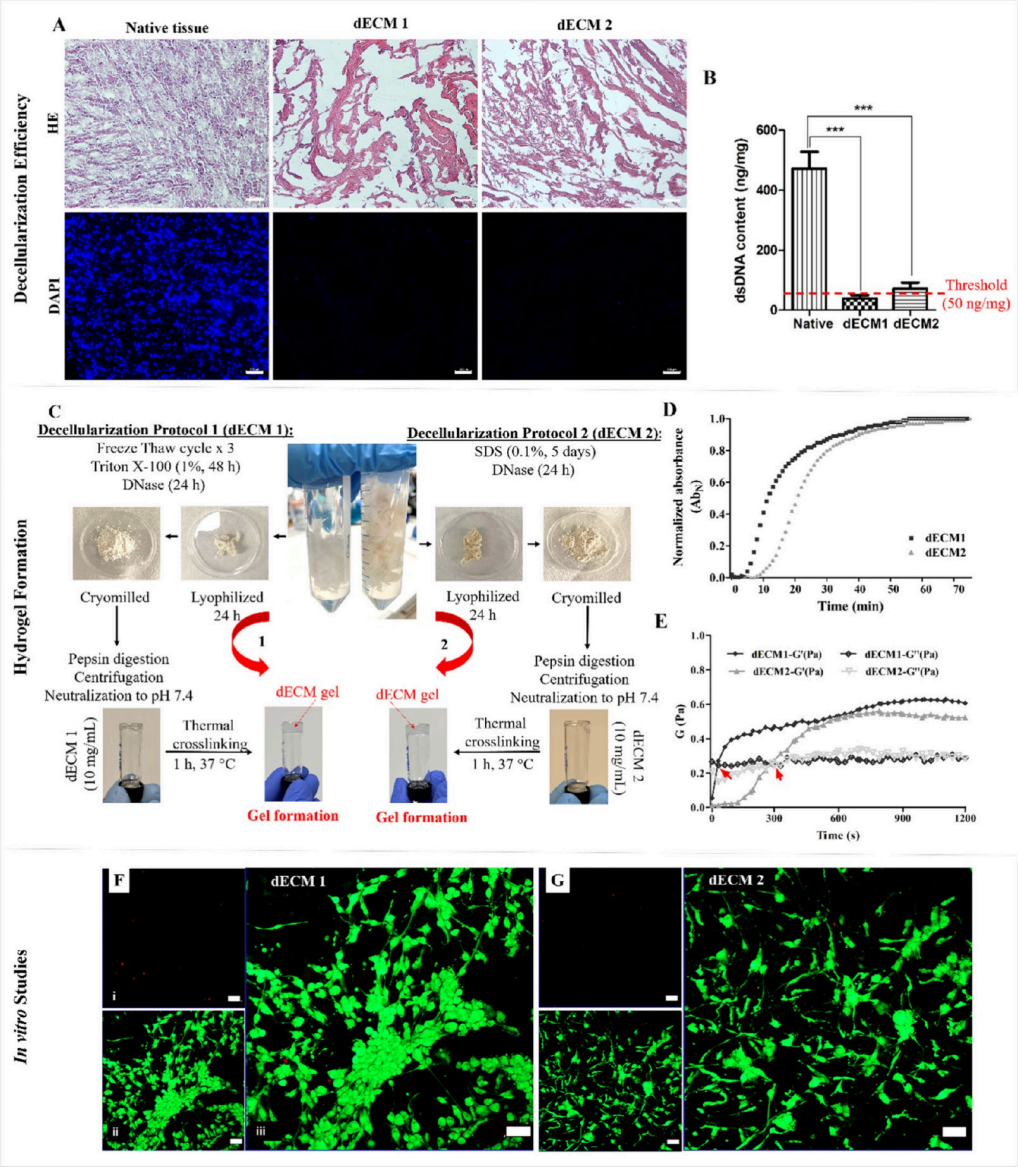
Decellularization of bovine brain tissue using 2 different protocols. Protocol 1 (dECM 1) consist of freeze thaw cycles combined with Triton X-100 and DNase treatment, Protocol 2 (dECM 2) is based on SDS and DNase treatments. (A) HE and DAPI images of native brain tissue and dECMs. (B) dsDNA amount (per mg sample). (C) Steps of the hydrogel formation from dECMs, (D) Turbidimetric gelation kinetics of dECM hydrogels. (E) Single frequency sweeps of dECMs at 37 °C. Red arrows indicate the cross over point where gelation begins. Black: dECM1, gray: dECM2. (F, G) Live–Dead cell analysis of U87 cells entrapped in dECM 1 and dECM 2 hydrogels, Day 7. CLSM images of (i) the dead cells (stained with ethidium homodimer-1, red), (ii) the live cells (stained with calcein AM, green) and (iii) merged images. Scale bar: (A) 100 μm, (F, G) 50 μm. Results are shown as means ± SD. Statistical analysis was carried out using unpaired *t* test. ***p < 0.001.

The residual dsDNA content in the dECMs and native brain tissues was determined (Figure 1B). According to the established decellularization efficiency criteria, the residual DNA content must be below 50 ng/mg of tissue.^16^ dECM1 contained 39.0 ± 10 ng/mg of DNA, whereas dECM2 contained 72 ± 20 ng/mg. Although both protocols significantly reduced DNA content, Protocol 1 demonstrated successful dsDNA removal and met the decellularization efficiency criterion.

The dECMs were compared for their retention of sulfated glycosaminoglycans (sGAGs), and analysis revealed differences in sGAG retention between the two protocols, where dECM1 and dECM2 containing 5.08 ± 0.35 and 4.07 ± 0.03 μg/mg sGAG ( in Supporting Information). The total protein content in native tissue, dECM 1, and dECM 2 was 200.5 ± 1.1 μg/mg, 252.1 ± 2.4 μg/mg, and 186.7 ± 8.8 μg/mg, respectively ( in Supporting Information), showing that dECM1 was better for retaining proteins. SDS-PAGE analysis was performed to evaluate protein profiles. The results showed protein bands spanning a range of molecular weight regions in both dECMs; however, dECM 1 exhibited more distinct and prominent bands than dECM 2, suggesting that dECM 1 is better than dECM 2 in terms of both protein retention and profile ( in Supporting Information). In order to complement these findings, a proteomic analysis was conducted on dECM1 using LC-MS/MS. The analysis confirmed the presence of several ECM-related proteins (), including laminin subunits and collagen type VI chains, which are relevant components of brain and glioblastoma microenvironments. Although fibronectin was not detected in the data set, this may be attributed to its low presence in decellularized brain tissue or potential losses during sample preparation steps such as FASP. Gene ontology enrichment analysis indicated that several proteins detected are involved in ECM-related functions, such as structural organization of the matrix and collagen interactions, supporting the relevance of dECM1’s protein content to the native extracellular matrix. This finding is supported by earlier studies demonstrating that Triton X-100 preserves ECM components better, whereas SDS treatment often results in loss of GAGs and critical structural proteins.^17^

In order to determine the presence of ECM components, native brain tissue and dECMs were further analyzed using FTIR. The preservation of ECM components is critical for successful decellularization. The FTIR spectra of both native tissue and dECMs displayed similar peaks in the peptide bond regions, confirming the retention of proteins during decellularization ( in Supporting Information). Amide bands associated with protein structure were present in the FTIR spectra including Amide I band (1700–1600 cm^–1^), Amide II band (1575–1480 cm^–1^), Amide III band (1350–1220 cm^–1^), and Amide A band (3500–3300 cm^–1^).^18^ Compared to native brain tissue, lipid associated bands, including CH_2_ and CH_3_ vibrations at 2800–3000 cm^–1^ and ester carbonyl (C = O) stretching at 1740 cm^–1^, were absent in both dECMs, demonstrating the effective removal of lipid components.

### Gelation of dECM Hydrogels and Cell Behavior within these Hydrogels

The dECMs obtained were enzymatically solubilized and formed hydrogels after 1 h of incubation at 37 °C (Figure 1C). The gelation kinetics of the dECM hydrogels were assessed by turbidimetric analysis. The results showed that dECM 1 underwent gelation almost twice as fast as dECM 2, with a slope of 0.0770, compared to 0.0391 (Figure 1D). dECM 1 displayed faster gelation kinetics in both the early and later stages of gelation. The rapid gelation observed in dECM1 was likely a result of the preserved structural proteins.^19,20^ The mechanical properties of the dECM hydrogels were evaluated by rheological single frequency testing at 37 °C to initiate gelation. Gelation was assumed to begin when the storage modulus (G’) surpassed the loss modulus (G’’), indicating the formation of a stable hydrogel (Figure 1E). For dECM 1, this crossover point occurred at 30 s, which was earlier than that for dECM 2 (600 s). In the steady-state phase, dECM 1 exhibited a higher elastic modulus (G’) of 4.17 ± 0.06 Pa and a viscous modulus (G’’) of 1.95 ± 0.04 Pa, reflecting greater mechanical stiffness. In contrast, dECM 2 showed a lower elastic modulus (G’) of 3.39 ± 0.05 Pa, while its viscous modulus (G’’) was similar at 1.97 ± 0.04 Pa. Overall, dECM 1 demonstrated faster gelation with superior mechanical stiffness than dECM 2, suggesting more rapid and robust network formation. Our results support previous reports highlighting that Triton X-100-treated scaffolds exhibit higher mechanical stiffness due to minimal collagen disruption compared to SDS-treated scaffolds, which results in a weakened ultrastructure.^20^ This mechanical robustness is particularly critical for applications requiring long-term structural stability. These results highlight the potential of dECM 1 for applications that require quicker gelation and enhanced mechanical properties, making it particularly suitable for 3D bioprinting and related applications.

Viability of glioblastoma cells entrapped in dECM hydrogels was assessed using Live–Dead analysis (Figure 1F, G). On Day 1, over 90% of the cells were alive across all hydrogels, indicating high initial cell viability. Branched and elongated cells were observed in dECMs, with more elongated cells in dECM 1 than in dECM 2. By Day 7, the cell viability remained above 90%, demonstrating that the cells could survive for at least 7 days postentrapment in the hydrogels. The presence of various ECM molecules in the dECM hydrogels support integrin-mediated cell adhesion, contributing to cell viability. The elongated and branched morphologies of the cells further confirmed their interactions with the dECM. By Day 7, the cells had spread and formed cellular networks within the hydrogels. In dECM 1, cells were primarily clusters, with most exhibiting a branched morphology and interacting with neighboring cells. Although similar cellular networks and branched morphologies were observed in dECM 2, the organization around large cell clusters were less in dECM 2. These findings suggest that dECM 1 provides a more favorable microenvironment for cell adhesion and organization compared to dECM 2.

Consequently, dECM 1 was selected for use in further studies because of its superior decellularization, preservation of biological components, superior gelation kinetics and mechanical strength, and enhanced compatibility with cells.

### Formation and Characterization of HAMA/dECM Hydrogel

In this study, a composite hydrogel model incorporating dECM and HAMA was developed. For this reason, HAMA was synthesized, and the degree of methacrylation (DM) was determined using the ^1^H NMR spectra of the HA and HAMA samples. ^1^H NMR spectroscopy confirmed the methacrylation of hyaluronic acid, as evidenced by the appearance of methacrylate peaks at 6.1, 5.6, and 1.85 ppm ( in Supporting Information) which was calculated to be 54%. This result indicates the incorporation of methacrylic groups at half of the positions on the HAMA molecule. The methacrylation reaction was further confirmed by FTIR spectroscopy ( in Supporting Information). The peak at 1730 cm^–1^, corresponding to the stretching vibration of C = O, confirmed the formation of ester bonds arising from the reaction between the carboxyl groups in hyaluronic acid and methacrylic anhydride. In particular, the peak at 1640 cm^–1^, representing the stretching vibration of C=C, confirmed the presence of methacrylate double bonds introduced during the reaction.

HAMA/dECM solutions were prepared and exposed to UV (365 nm, 1.6 J/cm^2^) for varying durations (1, 2, 3, and 4 min). The results revealed that 1 min of UV exposure was insufficient to achieve cross-linking of the pregel solution, whereas exposure for 2 min or more resulted in cross-linked hydrogels ( in Supporting Information). The hydrogels reached equilibrium swelling after approximately 24 h. The water contents of the hydrogels were calculated as 95.5% ± 0.3 for HAMA, 93.3% ± 0.3 for HAMA/dECM, and 90.2% ± 0.5 for dECM, respectively. Additionally, the degree of swelling (DS) was determined to be 2152% ± 140 for HAMA, 1394% ± 60 for HAMA/dECM, and 1394% ± 241 for dECM hydrogels, respectively, highlighting their high water retention capacities. GBM tissue is characterized by significantly higher water retention than healthy brain tissue. This is due to disruption of the blood-brain barrier, leading to vasogenic edema, abnormal vascularization, and overexpression of aquaporin channels such as AQP4, which facilitate water movement.^21^ These factors collectively create a hyperhydrated microenvironment in GBM. The hydrogels, with water contents of up to 95.5%, effectively mimicked the elevated water retention of GBM tissue.

The elastic modulus ( *G′* ) and viscous modulus ( *G″* ) were measured as functions of strain (γ*) to evaluate the viscoelastic properties of the hydrogels (Figure 2A). Across all samples, a linear viscoelastic region (LVER) was observed, where both *G′* and *G″* remained constant with increasing strain, demonstrating structural stability. The dominance of *G′* over *G′*′ in all the samples confirmed that the hydrogels were predominantly elastic. Among the hydrogels, 1H3D exhibited superior viscoelastic behavior, with the highest *G′* (458.30 ± 13.39 Pa) and *G*″ (65.17 ± 6.46 Pa) values and robust structural integrity even at higher strains. In contrast, HAMA hydrogels with a lower dECM content and dECM hydrogels alone demonstrated significantly reduced stiffness and elasticity (Figure 2A, B). GBM tumors exhibit distinct viscoelastic properties, displaying both solid- and fluid-like behaviors.^22^ The elastic modulus (*G′*) and viscous modulus (*G″*) of GBM tumors have been reported to be 300 < *G*′<1200 Pa and 20 < *G*″<120 Pa, respectively.^23,24^ Because the 1H3D hydrogel falls within this reported range, this suggests that 1H3D is more effective in mimicking the mechanical properties of GBM tumors.

**Figure 2:**
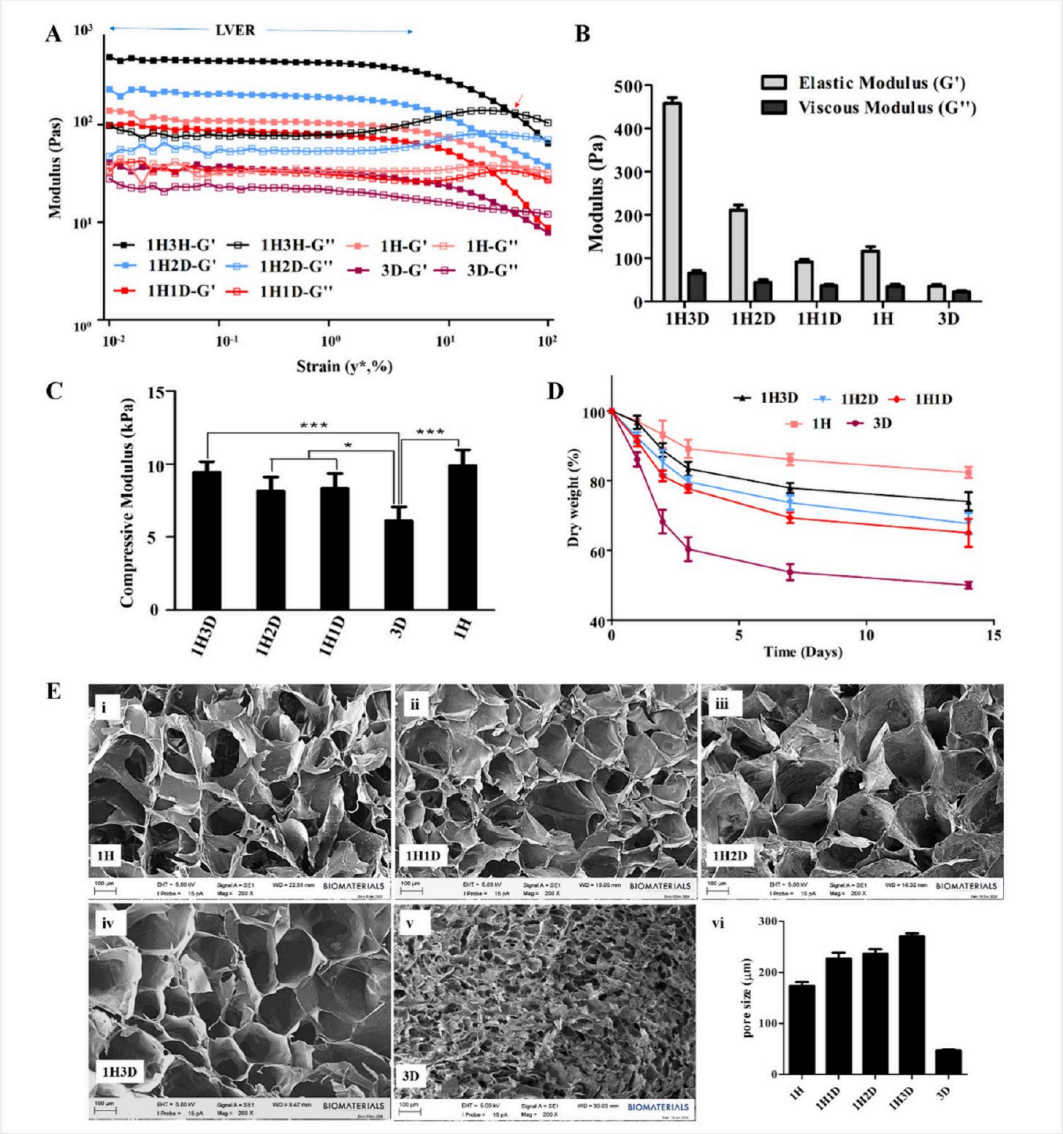
Characterization of the HAMA/dECM (H/D) composite hydrogels. (A) Strain sweep test: the elastic modulus (*G′*) and viscous modulus (*G″*) as a function of strain (γ*) at constant frequency and controlled oscillatory shear strain. Blue arrow: Linear viscoelastic region (LVER). Red arrow: Transition point where *G″* = *G′*. (B) The elastic and viscous modulus of the hydrogels at stationary (LVER). (C) Compressive modulus of the hydrogels. (D) Degradation profiles of the hydrogels. Results are shown as means ± SD, comparisons tests were performed by one-way ANOVA with Bonferroni’s posthoc. *p < 0.05, **p < 0.01, and ***p < 0.001. (E) The scanning electron microscope (SEM) image of the hydrogels. (i) 1H, (ii) 1H1D, (iii) 1H2D, (iv) 1H3D, (v) 3D, and the graph shows the average pore size of the hydrogels. Scale: 100 μm.

Compression test was also conducted to determine the mechanical properties of the hydrogels, and the compressive moduli of the 3D, 1H, 1H1D, 1H2D, and 1H3D hydrogels were determined as 6.11 ± 0.94, 9.91 ± 1.06, 8.15 ± 0.95, 8.33 ± 1.02, and 9.44 ± 0.73 kPa, respectively (Figure 2C). These results indicated that the incorporation of HAMA significantly increased the compressive modulus of the hydrogels. While there was no significant difference in the compressive moduli between the 1H1D and 1H2D hydrogels, increasing the dECM component to 3% (w/v) significantly increased the compressive modulus, demonstrating a synergistic relationship between dECM and HAMA. Postcompression analysis revealed notable structural differences between the hydrogels. Fracturing occurred in 1H hydrogels, whereas fluid diffusion was dominant in 3D hydrogels. However, the composite hydrogels maintained their structural integrity without visible deformations, indicating strong molecular interactions. The hybrid nature of the HAMA/dECM hydrogels likely provides a robust matrix with high flexibility, replicating glioblastoma tissue, as supported by recent reports.^25,26^ Studies have reported higher stiffness values in high-grade GBM.^27–29^ Healthy brain tissue typically exhibits a softer and more uniform mechanical profile, with a Young’s modulus of approximately 1 kPa.^6,7^ In contrast, high-grade gliomas exhibit much stiffer properties, with values around 11.4 ± 4.9 kPa.^8^ Both tumor cells and the ECM contribute to solid stress within the GBM microenvironment, impairing vascular and lymphatic systems and leading to hypoxia and immune evasion, underscoring the importance of the biomechanical properties of the ECM. The 1H3D hydrogel, with compressive moduli close to those of GBM tumors, highlights its potential for replicating the biomechanical environment of the GBM extracellular matrix.

The degradation profiles of the hydrogels were determined (Figure 2D), and the highest weight loss was observed with dECM hydrogels, especially in the first 2 days (up to 35% for dECM), and it was lower in the following periods. The lowest degradation was observed in HAMA hydrogels, followed by HAMA/dECM hydrogels. These findings align with previous reports indicating that dECM hydrogels degrade more rapidly because of their reliance on noncovalent interactions, which provide limited long-term structural integrity.^30^ In contrast, HAMA hydrogels degrade more slowly owing to the formation of covalent bonds, which results in enhanced mechanical stability and durability.^31^ After decellularization and pepsin digestion, dECM at neutral pH forms a hydrogel at 37 °C through hydrogen bonding, hydrophobic interactions, and electrostatic forces. These bonds provide strong structural integrity, with hydrogen bonds between the hydroxyl and amine groups in dECM proteins, enhancing stability and hydrophobic interactions at physiological temperatures, thus increasing mechanical strength.^31^ However, these interactions are inherently weaker than the covalent bonds in HAMA hydrogels, which are formed between methacrylate groups during UV cross-linking, creating a highly stable network.^32^ In cell culture applications, the degradation rate of hydrogels is critical for optimizing cell growth and tissue formation. Hydrogels with high degradation rates provide a quick release of bioactive factors, such as growth factors, cytokines, and other signaling molecules, and space for cells to expand, but lack the mechanical integrity needed for sustained support. In contrast, hydrogels with low degradation rates offer prolonged support and a stable environment but impede tissue remodeling and integration.^33^ HAMA/dECM hydrogels demonstrated an intermediate degradation profile, striking a balance between bioactive factor release and structural integrity. While the dECM in the composite hydrogels offered excellent biocompatibility and mimicked natural tissue environments by reflecting biological composition, HAMA provided superior mechanical strength and durability owing to its slower degradation rate.

The hydrogels were examined using SEM, and all hydrogels (1H, 1H1D, 1H2D, 1H3D, and 3D) exhibited a highly porous 3D network structure (Figure 2E). The dECM hydrogels had smaller average pore sizes (98 ± 37 μm) than the HAMA-based hydrogels. The average pore sizes of the HAMA-based hydrogels were 171 ± 24 μm for 1H, 227 ± 42 μm for 1H1D, 236 ± 33 μm for 1H2D, and 270 ± 37 μm for 1H3D. The porous and interconnected network structures observed in these hydrogels provide a favorable microenvironment for the survival and expansion of loaded cells, making them highly suitable for applications in tissue engineering and cell culture.

### Viability and Behavior of Cells within the HAMA/dECM Hydrogel

U87 and HMC3 cells were entrapped within the 1H3D hydrogel, and their viability was evaluated on days 1, 3, 7, and 14 using Live–Dead staining and the Alamar Blue viability assay. Live–Dead analysis results showed that the viability of U87 cells (Figure 3A) was 93.7%, 93.2%, 97.4%, and 98.2% on days 1, 3, 7, and 14, respectively, whereas HMC3 cell viability was 92.5%, 94.4%, 98.4%, and 99.1%, respectively. These high viability levels indicated that the hydrogel provided a supportive environment for cell survival and growth for at least 14 days. Both U87 and HMC3 cells branched and elongated within the HAMA/dECM hydrogels. U87 cells displayed a spindle-like shape with long extensions, forming connections with neighboring cells. HMC3 cells also became elongated and fibroblast-like by day 7. Additionally, an increase in cell proliferation was observed, as reflected by the metabolic activity measured using the Alamar Blue assay (Figure 3B). U87, HMC3, and coculture conditions all demonstrated an upward trend in cell percentage over time, indicating consistent growth and proliferation. Although U87 and HMC3 cells exhibited relatively similar growth profiles with steady increases, the coculture condition showed higher cell percentages by day 14. This finding suggests supportive interactions between U87 and HMC3 cells, which enhance both cell growth and survival in the coculture environment.

**Figure 3:**
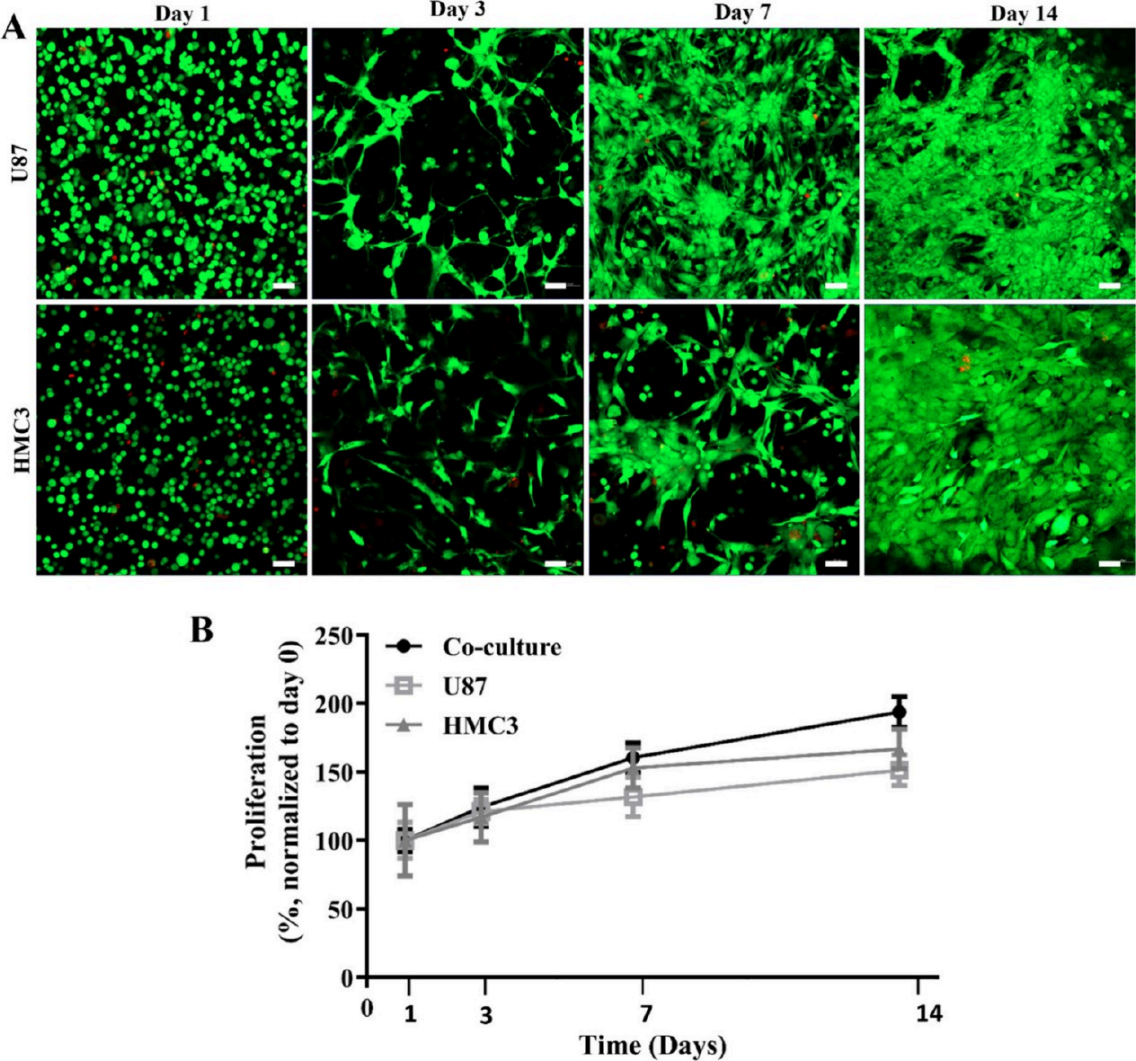
Viabilities of the cells. (A) Live–dead analysis of U87 and HMC3 cells for 14 days. The dead cells (stained with ethidium homodimer-1, red), the live cells (stained with calcein AM, green). Scale bar: 50 μm. (B) Alamar Blue assay shows proliferation of cells in the 1H3D hydrogel. Results are shown as means ± SD.

The morphology of U87, HMC3, and U87/HMC3 cells was analyzed using Phalloidin and DAPI staining to visualize the actin filaments and nuclei, respectively (Figure 4). Staining of cells loaded into the 1H, 3D, and 1H3D hydrogels were performed. In the 1H hydrogel, the cells exhibited limited spreading, with actin filaments primarily localized around the nuclei. The rounded cell shape and lack of significant spreading indicated weak interactions between the cells and the matrix. In both the 3D and 1H3D hydrogels, U87 cells displayed a highly organized network of actin filaments with prominent stress fibers, suggesting a robust cytoskeletal structure that supports motility and mechanical stability. Conversely, HMC3 cells demonstrated more diffuse and less organized actin filaments in both the 3D and 1H3D hydrogels, indicating lower cytoskeletal organization. The coculture of U87 and HMC3 cells revealed a mixed pattern of actin filament organization. This included regions with well-defined stress fibers, which are characteristic of U87 cells, and regions with more diffuse actin structures, similar to those observed in HMC3 cells.

**Figure 4:**
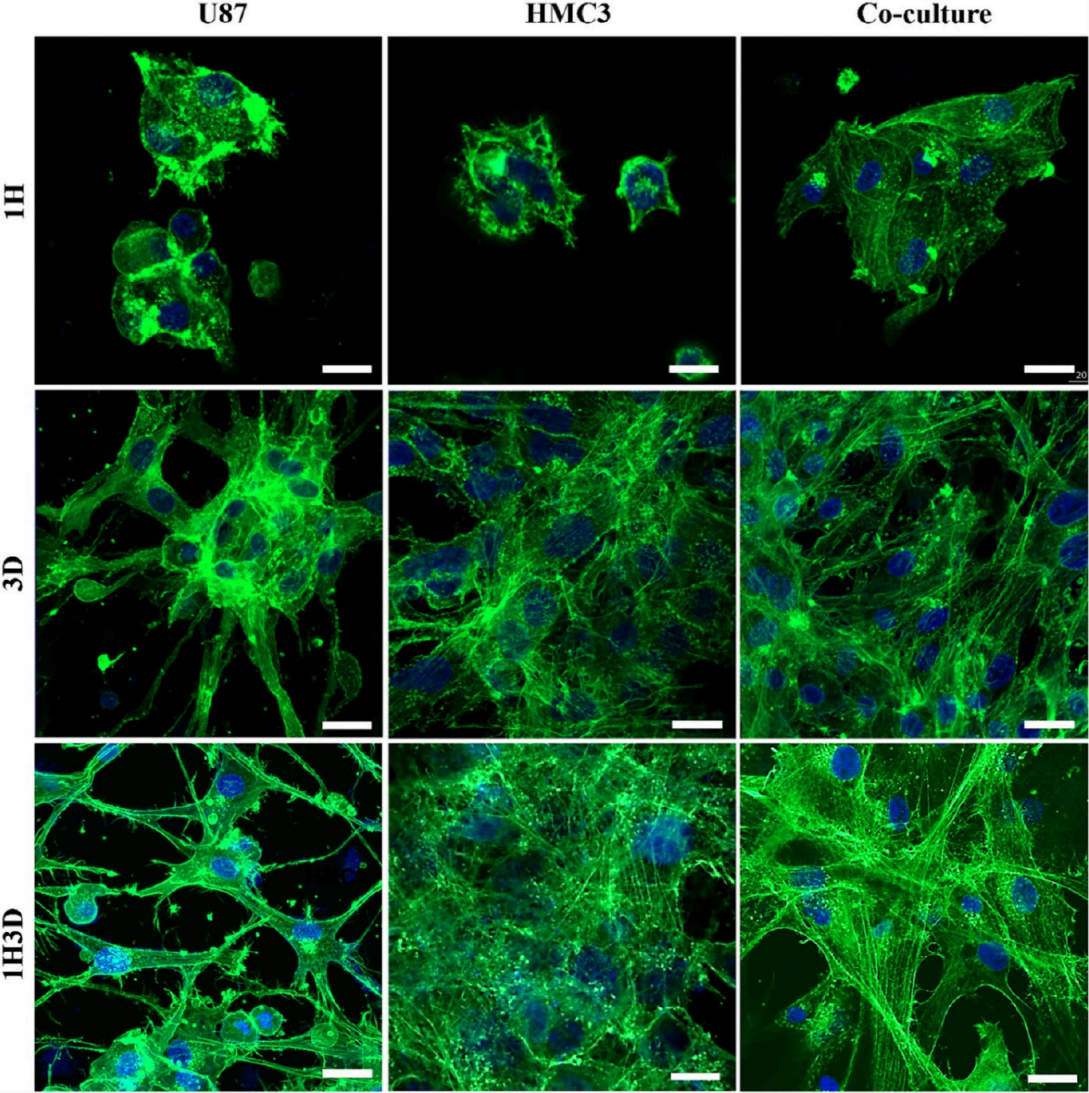
CLSM micrographs of U87, HMC3, and U87/HMC3 coculture cells loaded in 1H, 3D, and composite hydrogels. F-actin filaments (phalloidin; green), nucleus (DAPI: blue). Scale bar: 20 μm.

### Invasion of Glioblastoma Cells within the HAMA/dECM Hydrogel

Glioblastoma tumors are characterized by their highly invasive nature, which enables GBM cells to infiltrate healthy brain tissues. This invasive capacity makes complete surgical resection impossible and contributes significantly to tumor recurrence. An invasion assay was conducted using EGFP-labeled U87 spheroids to assess the invasive potential of GBM cells. Images were obtained at 0, 24, and 48 h, and the invasion distances of the cells from the edge of the spheroids were measured to determine their invasion potential within the hydrogels. In the HAMA hydrogel, U87 cells exhibited no detectable invasion (Figure 5A, B), whereas in the 1H3D hydrogel, the cells demonstrated pronounced invasion, suggesting that the composite hydrogel promoted matrix remodeling and provided an environment that was more conducive to cell migration. Furthermore, when U87 cells were cocultured with microglia in the composite hydrogel, their invasion distances were greater (41%) than those observed in monoculture conditions. The invasive behavior of GBM is driven by intrinsic cellular properties and the extracellular microenvironment, which includes stromal cells such as microglia and the extracellular matrix.^34^ The findings underscore the role of microglia in promoting GBM invasion, potentially through the secretion of pro-invasive factors or direct cell–cell interactions. Quantitative gene expression analysis revealed that coculturing U87 cells with microglia within the hydrogel platform resulted in the upregulation of genes associated with extracellular matrix remodeling and tumor invasiveness. Specifically, expression levels of MMP-2 and MMP-9 were higher in the coculture condition compared to U87 monocultures (Figure 5C). Although this upregulation does not directly confirm proteolytic activity, it suggests that microglia may contribute to a signaling environment that promotes ECM degradation and enhances glioblastoma cell invasiveness. These findings provide preliminary molecular evidence that the hydrogel model supports microenvironmental interactions relevant to dynamic ECM remodeling in GBM. Furthermore, the viscoelastic properties and biochemical composition of hydrogels are critical for imitating the tumor microenvironment and influencing GBM invasion. Studies have shown that stiffer hydrogels with higher *G′* values enhance focal adhesion formation, cytoskeletal contractility, and cell motility.^23,35,36^ Beyond their mechanical strength, 1H3D also incorporates biochemical signals, including integrin-binding motifs, which promote cell adhesion and invasion. These biochemical cues facilitate cytoskeletal remodeling and matrix metalloproteinase (MMP)-mediated ECM degradation, enabling GBM cells to effectively invade the surrounding environment. In contrast, HAMA hydrogels lack the biochemical signals required to support tumor invasion. This comparative analysis demonstrated that 1H3D hydrogels are better suited for mimicking the biomechanical and biochemical complexity of the GBM tumor microenvironment.

**Figure 5:**
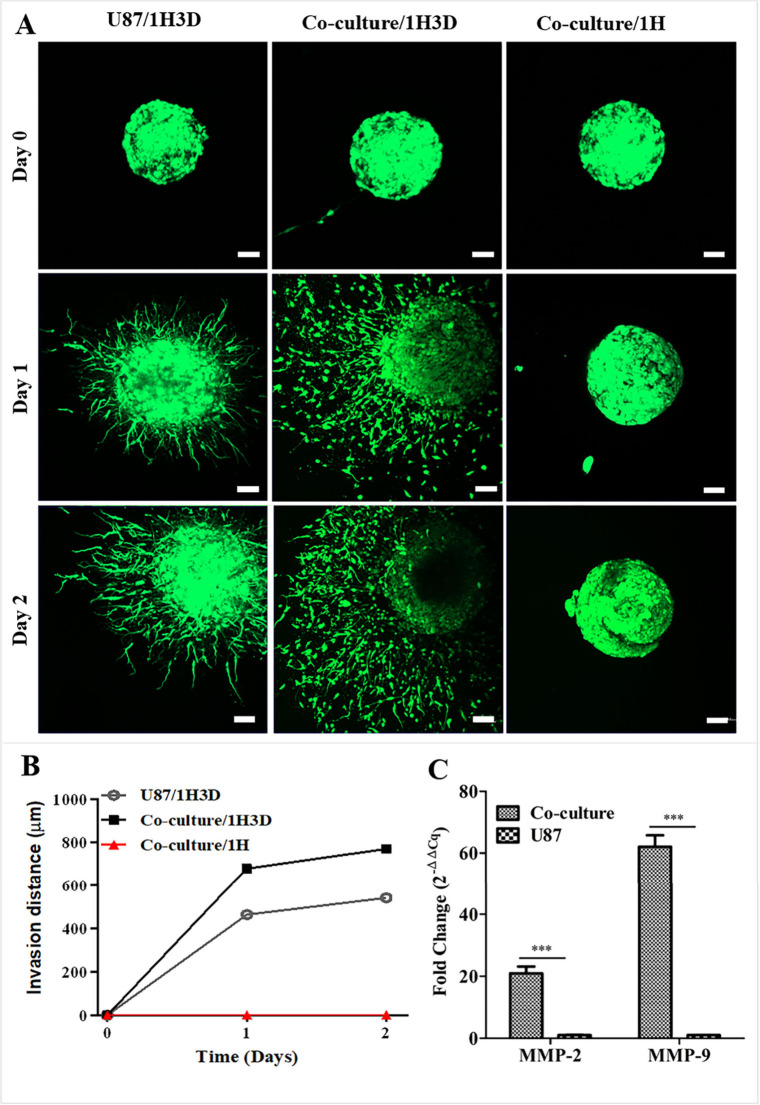
Cell invasion in the hydrogels. (A) CLSM images of the glioblastoma cell invasion. Scale: 1 mm. (B) Invasion distances monitored for 2 days. (C) Gene expression analysis of the cells cultured in 1H3D hydrogels. Data are presented as fold change (2^–ΔΔ*Cq*^) relative to U87 monoculture, ***p < 0.001.

### Proliferation of Cells within the HAMA/dECM Hydrogel

U87, HMC3, and U87/HMC3 cells were cultured in 1H3D hydrogels for 7 days, followed by immunofluorescence staining using *K*_i_-67 antibody (Figure 6). The results demonstrated that both U87 and HMC3 cells expressed *K*_i_-67 protein, indicating active cell proliferation in the 1H3D hydrogel environment. Notably, the coculture of the cells exhibited higher *K*_i_-67 expression than monocultures, indicating enhanced proliferation in the coculture environment. This phenomenon is likely attributable to synergistic interactions between glioblastoma and microglia cells. Microglia play a key role in glioblastoma progression by secreting growth factors, cytokines, and other signaling molecules that promote glioblastoma cell proliferation and invasion.^37^ The coculture condition likely facilitates bidirectional communication through these signaling pathways, creating a dynamic microenvironment that stimulates cellular activity and proliferation. The ability of microglia to support tumor growth through paracrine signaling and direct cell–cell interactions underscores the complex interplay between glioblastoma cells and their surrounding microenvironment. Various mechanisms through which microglia enhance GBM cell proliferation have been demonstrated.^38^ In the GBM microenvironment, microglia acquire tumor-supportive phenotypes and adopt a protumoral role. These results are consistent with those of previous studies highlighting the importance of tumor-stroma interactions in glioblastoma.^39,40^

**Figure 6:**
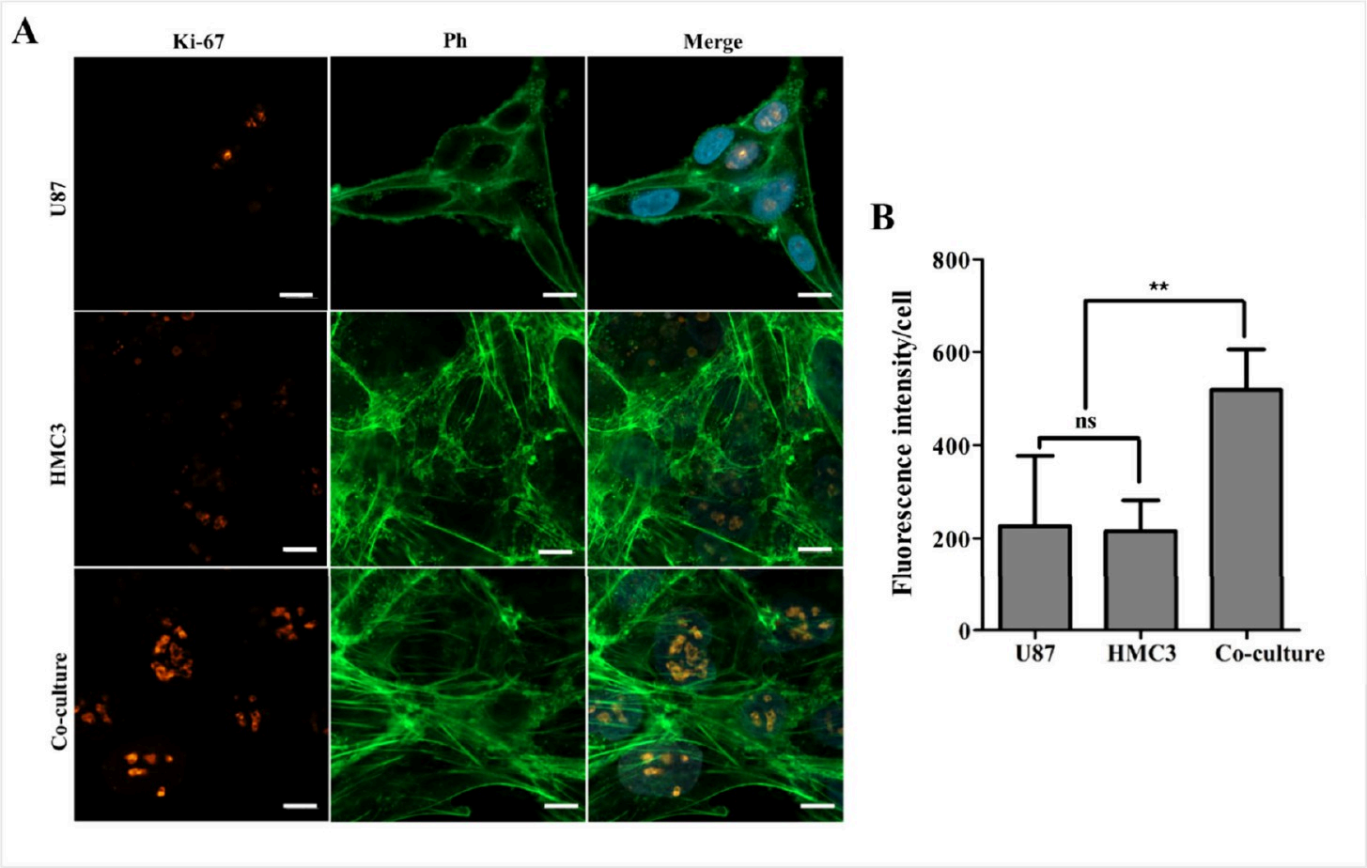
*K*_i_-67 expression of the cells. (A) CLSM images of immunofluorescence staining of U87, HMC3, and coculture cells for the expression of proliferation marker *K*_i_-67. (Green: F-actin, phalloidin, red: *K*_i_-67, blue: nucleus, DAPI). Scale bar: 20 μm. (B) The graph of fluorescence intensities per cell. Results are shown as means ± SD, comparisons tests were performed by one-way ANOVA with Bonferroni’s posthoc. ns: not significant, **p < 0.01.

In order to further investigate the effects of microglia on glioblastoma, proteomic analysis was performed comparing U87 cells cultured alone and in coculture with microglia (HMC3). The comparative analysis revealed differential expression of several proteins associated with metabolic adaptation, chromatin organization, and immune-related functions. Gene ontology enrichment analysis of the coculture condition showed over representation of functional categories such as oxygen binding, carbon dioxide transport, peroxidase activity, nucleosomal DNA binding, and chromatin remodeling (), which may reflect an altered cellular state in response to microglia-associated cues. These categories suggest that microglia may induce transcriptional and metabolic shifts in glioblastoma, potentially reflecting adaptive responses to a more inflammatory microenvironment. Additionally, enrichment of terms related to immune modulation suggests that paracrine signaling may contribute to shaping the tumor phenotype in coculture. Although the current study does not directly assess cytokine levels or specific intracellular signaling pathways such as NF-κB or MAPK, these findings offer preliminary insight into the molecular changes associated with tumor–microglia interactions and might offer a basis for future studies aimed at elucidating the specific signaling mechanisms involved.

Although the 1H3D hydrogel combined with glioblastoma–microglia coculture mimics the some characteristics of the GBM microenvironment, certain limitations remain. First, the use of immortalized cell lines instead of patient-derived glioblastoma or microglial cells reduces the platform’s ability to represent patient-specific variability and tumor behavior. Second, while microglia were included as a key stromal component, other important cell types such as astrocytes, endothelial cells, and lymphocytes were not incorporated. These cells play essential roles in shaping the immunosuppressive, angiogenic, and invasive properties of GBM tissues. The lack of these additional components limits the ability of the platform to represent the cellular diversity and complexity of the *in vivo* microenvironment. This platform replicates the glioblastoma microenvironment to a certain extent by capturing essential features such as ECM composition, biomechanical properties, and tumor–microglia interactions, while lacking certain components like hypoxia gradients which play a central role in GBM heterogeneity and treatment resistance. Incorporation of hypoxic conditions remains an important aspect for future refinement of the model. Future studies should therefore integrate these elements to achieve a more comprehensive and biologically accurate setting. Moreover, although this study primarily focused on proliferation and invasion, further investigation of the molecular mechanisms activated under coculture conditions would provide deeper insights. Multiomic profiling could help identify key signaling pathways or gene expression profiles involved in GBM–microglia interactions in the hydrogel platform. Understanding cellular responses to therapeutic agents within the platform is essential for establishing its translational relevance.

In conclusion, the 1H3D hydrogel and glioblastoma–microglia coculture system offers a relevant and practical approach for *in vitro* investigation of the GBM microenvironment. Its ability to reflect both the physical characteristics and biochemical composition of GBM tissue, while enabling dynamic interactions between tumor and stromal cells, highlights its potential for advancing glioblastoma research and supporting therapeutic development.

## Experimental Section

### Preparation and Characterization of Decellularized ECM

#### Decellularization of Brain Tissue (dECM)

Fresh bovine brains were obtained from a local slaughterhouse and processed immediately to ensure tissue integrity. Prior to decellularization, the tissues were thoroughly washed with phosphate buffered saline (PBS, 10 mM, pH 7.4) supplemented with 1% penicillin/streptomycin at 4 °C for 2 h to reduce contamination. Subsequently, the brains were cut into uniform pieces (0.5 × 0.5 × 0.5 cm^3^) to facilitate efficient decellularization. Bovine brain tissue was decellularized using two different protocols to remove cellular components while preserving the extracellular matrix (ECM). The control samples were fixed in 10% formalin at room temperature for 24 h. The first decellularization protocol involved freeze–thaw cycles and Triton X-100 treatment. Brain tissues were frozen in PBS (10 mM, pH 7.4) at −80 °C for at least 5 h and thawed at room temperature. The freeze–thaw cycle was repeated three times. The samples were stirred in distilled water containing 1% penicillin-streptomycin for 24 h and immersed in 1% Triton X-100 (in 10 mM Tris-HCl, 1 mM EDTA, pH 7.5), and stirred at 80 rpm for 48 h. Samples were washed in dH_2_O containing 1% penicillin-streptomycin for 24 h with several solution changes. In order to eliminate residual DNA, tissues were treated with 40 U/mL DNase in 10 mM MgCl_2_ buffer (pH 7.5) for 24 h. The resultant dECM was washed with dH_2_O 10 times, with centrifugation after each wash. In order to assess the decellularization efficacy, small samples were fixed in 10% formalin at room temperature for 24 h.

In the second protocol, brain tissues were stirred in 0.1% sodium dodecyl sulfate (SDS) solution for 4 days, then washed with dH_2_O for 5 h, and treated with DNase (40 U/mL in 10 mM MgCl_2_ buffer) for 24 h, followed by 10 cycles of washing with dH_2_O, including centrifugation between each wash. After washing, the dECM samples were frozen at −80 °C overnight and lyophilized for 24 h.

#### Assessment of Decellularization Efficiency

The decellularization methods were evaluated based on the removal of cell nuclei, reduction of the remaining DNA content, and elimination of lipids. Formalin-fixed native and decellularized brain tissues were stained with 4′,6-diamidino-2-phenylindole (DAPI) and hematoxylin and eosin (H&E) to detect residual cellular components. Double-stranded DNA (dsDNA) quantification was performed to measure residual DNA, and samples with concentrations below 50 ng/mg were classified as successfully decellularized.^16^

In order to determine the efficiency of decellularization in removing dsDNA residues from the tissue, DNA was isolated using the DNeasy Blood & Tissue Kit (Qiagen) according to the manufacturer’s instructions. Briefly, tissue samples were lysed, and the lysates were passed through spin columns to purify DNA. The concentration of dsDNA was measured using a NanoDrop spectrophotometer (Thermo Scientific NanoDrop One) (280 nm).

Fixed tissue samples were embedded in OCT compound (Tissue-Tek) and frozen at −80 °C. Cryosections (5 μm) were obtained using a cryomicrotome (Leica, Cryomicrotome CM1520) and mounted on slides. In order to assess the efficacy of decellularization in removing cellular residues, the sections were stained with H&E according to the literature^41^ and DAPI (0.5 μg/mL in PBS) at room temperature for 15 min. The stained tissue sections were examined under a light microscope (Zeiss Axio Imager M2) for histological evaluation.

#### sGAG Staining of Decellularized Samples

In order to quantify sulfated glycosaminoglycans (GAGs) left behind after decellularization, 1,9-dimethylmethylene blue (DMMB) staining was performed.^42^ The GAG content was measured using a solution containing 40 μM DMMB in 0.3% glycine (w/v), 0.16% sodium chloride (w/v), and 0.01 M acetic acid. The samples were diluted with the working solution at a 1:10 ratio, and the absorbance was measured at 525 nm using a spectrophotometer (PerkinElmer, 8500FL). The GAG concentrations were calculated using a standard curve generated from known concentrations of chondroitin sulfate.

#### Protein Profile

Total protein content of dECMs after decellularization was measured using the Pierce BCA Protein Assay Kit (Thermo Fisher). The protein extracts were mixed with BCA reagent and incubated at 37 °C for 30 min, the absorbance was measured at 562 nm. The total protein concentration was calculated using a standard curve prepared with known BSA concentrations. The protein profiles of the dECMs were examined by SDS-PAGE. The protein extracts were loaded into the wells of an acrylamide gel and run at 150 V for 1 h. After electrophoresis, the gel was stained with Coomassie blue, and the protein bands were visualized using the Bio-Rad Imaging System.

#### FTIR Analysis

The preservation of proteins in the brain samples following decellularization was determined using Fourier-transform infrared (FTIR) spectroscopy (Shimadzu, IR Tracer 100). Both the dECMs and native brain tissue samples were scanned in the 400–4000 cm^–1^ range. The resulting FTIR spectra were then compared.

#### Preparation of Soluble dECM

Lyophilized brain dECM samples were grounded into a fine powder using a cryomill. The powdered dECM (final concentration of 10 mg/mL) was enzymatically digested with pepsin (final concentration of 1 mg/mL in 0.1 M HCl) by stirring at 300 rpm at room temperature for 48 h. The dECM solutions were centrifuged at 4 °C at 13000 rpm for 10 min and neutralized to pH 7.4, by adding drops of cold NaOH (0.5 M). The solution was immediately frozen at −80 °C overnight and lyophilized for 24 h.^43,44^

#### Gelation Kinetics of dECM Hydrogels

The gelation kinetics of the dECM hydrogels were studied using a turbidity assay.^45^ dECM solutions (10 mg/mL in PBS) were prepared, and 100 μL of the solution was transferred into 96 well plates. The absorbance at 405 nm was recorded every 3 min for 90 min using a plate reader (PerkinElmer, 8500FL) at 37 °C. Absorbance values (A) were normalized to a scale of 0% (at time = 0 min) to 100% (at the time of maximum absorbance), using the following equation:

$$A_{N}=\frac{A_{t}-A_{0}}{A_{\max}-A_{0}}$$

where *A*_N_ is the normalized absorbance at a specific time, *A*_*t*_ is the absorbance at that time, and *A*_0_ and *A*_max_ are the initial and maximum absorbances, respectively.

#### Single Frequency Analysis

The gelation kinetics of the dECM hydrogels (10 mg/mL in PBS) were also analyzed using a rheometer (Kinetux, Malvern) at 37 °C. The measurements were performed at a constant frequency of 1.0 Hz with a strain amplitude within the linear viscoelastic region. The storage modulus (*G′*), loss modulus (*G″*), and phase angle (δ) were continuously recorded. Gelation was defined as the point at which *G′* exceeds *G″*, indicating a transition from a viscous to an elastic state.^46^

### Preparation and Characterization of Composite Hydrogels

#### Synthesis of Methacrylated Hyaluronic Acid

Hyaluronic acid was dissolved in dH_2_O at room temperature (1%, w/v), and dimethylformamide (DMF) was gradually added (2:3, DMF: H_2_O, v:v). The solution was incubated at 4 °C for 1 h. Methacrylic anhydride (final concentration of 3%, v/v) was added, and the solution was incubated overnight at 4 °C, maintaining a pH of 8–9.^47^ The solution was placed in a dialysis tube (CO 10 000 Da) and dialyzed against dH_2_O at 4 °C for 3 days. It was then lyophilized to form foam. The degree of methacrylation (DM) of methacrylated hyaluronic acid (HAMA) was calculated from the high-resolution ^1^H NMR spectra of hyaluronic acid and HAMA (400 MHz Bruker DPX 400). The degree of methacrylation, defined as the number of methacrylate groups per hyaluronic acid disaccharide, was determined by the ratio of the relative peak integrations of the methacrylate protons (6.1, 5.6, and 1.85 ppm) to the methyl protons present in the hyaluronic acid structure (1.9 ppm).^48^

#### Preparation of HAMA/dECM Hydrogel

The photoinitiator Irgacure 2959 was dissolved in DMEM (0.3%, w/v) at 37 °C and subsequently cooled to 4 °C. HAMA (final concentration of 1%, w/v) was added to this solution and stirred overnight at 4 °C. The dECM powder (final concentrations of 1%, 2%, and 3%, w/v) was then incorporated into the HAMA mixture and stirred overnight at 4 °C to achieve a uniform and homogeneous solution. The hydrogels were cross-linked using UV irradiation (365 nm, 0.160 j/cm^2^). The concentrations described above correspond to the final weight/volume (w/v) ratios of each component in the hydrogel formulations. In the naming system, ‘H’ refers to HAMA, and ‘D’ refers to dECM.

#### Equilibrium Water Content (EWC)

The equilibrium water content of the hydrogels was determined using the swelling properties of the hydrogel. The hydrogels were lyophilized, weighed, then incubated in PBS (10 mM, pH 7.4) at 37 °C until equilibrium, and weighed. The equilibrium water content% was calculated as follows:

$$\mathrm{EWC}\;\left(\%\right)=\frac{W_{s}-W_{d}}{W_{s}}\times 100$$

where *W*_s_ is the swollen weight and *W*_d_ is the dry weight.

#### Strain Sweep Testing

Strain sweep test (amplitude sweep) was conducted to determine the rheological properties of the hydrogels using a constant frequency of 1.0 Hz.^19^ Measurements were performed at room temperature using a rheometer (Kinetux, Malvern, United Kingdom), and *G′* and *G″* were recorded across a strain range of 0.01 to 100%, as a function of strain.

#### Mechanical Testing

In order to determine the mechanical properties of the hydrogels, a compression test was conducted. HAMA/dECM hydrogels (10 mm diameter, 5 mm height) were prepared and cross-linked with UV light (365 nm, 0.160 j/cm^2^). The hydrogels were incubated in PBS until the swelling equilibrium was reached (24 h). Compression tests were performed at room temperature using a mechanical testing device (Shimadzu AGS-X, Japan) at a compression rate of 0.1 mm/min. A stress–strain curve was plotted, and the compressive modulus of the hydrogels was determined from the initial slope of the curve.

#### Scanning Electron Microscopy

The internal structure of the dECM hydrogels was examined using a scanning electron microscope (Zeiss, Evo 10). The hydrogels were frozen at −80 °C and lyophilized. The samples were coated with a thin layer of gold (20 Å thickness, 120 s) under vacuum to enhance their conductivity. The samples were then imaged using SEM, and the pore sizes were measured from the images obtained using ImageJ software.

#### *In Situ* Degradation (in PBS)

Hydrogels were washed with distilled water and lyophilized to determine their initial dry weights (n = 3). They were then incubated in PBS (pH 7.4, 10 mM) at 37 °C for 14 days to study their degradation. On days 1, 2, 3, 7, and 14, the samples were removed, rinsed with dH_2_O, lyophilized, and weighed. The weight loss was calculated using the following equation:

$$\mathrm{Remaining weight}\;\left(\%\right)=\frac{W_{0}}{W_{t}}\times 100$$

where *W*_0_: initial dry weight, *W*_*t*_: dry weight at time *t*.

### *In Vitro* Studies

#### Cell Culture

U87 (ATCC, HTB-14) and HMC3 (ATCC, CRL-3304) cell lines were used as glioblastoma and microglia cell sources, respectively. They were cultured in T75 flasks with complete medium, high-glucose Dulbecco’s Modified Eagle’s medium (DMEM, Gibco) supplemented with 10% fetal bovine serum (FBS, Sartorius), and 1% penicillin/streptomycin (Gibco). The cells were incubated in a humidified incubator (37 °C, 5% CO_2_) with medium replaced every 2 days.

#### Cell Viability

The viability of U87 and HMC3 cells was determined on days 1, 3, 7, and 14 using Live–Dead and Alamar Blue viability assays (Thermo Scientific). For Live–Dead assay, the cells were stained with 2 μM calcein AM and 4 μM ethidium homodimer-1 in DMEM without phenol red for 30 min at 37 °C. They were then washed with PBS and examined by confocal laser scanning microscopy (CLSM, Zeiss LSM 900). ImageJ software was used to count the live and dead cells and calculate the viability percentages. For Alamar Blue assay, medium was replaced with Alamar Blue reagent (1:10, v/v, in complete medium) and incubated for 3 h at 37 °C. The end-point absorbance values were measured at 570 and 600 nm.

#### Cytoskeleton and Nuclei Staining of U87 and HMC3 Cells

Phalloidin and DAPI staining were performed to visualize the morphology of glioblastoma and microglia cells. After 7 days of incubation, the cells were fixed with paraformaldehyde (4%, w/v) for 15 min at room temperature. The cells were permeabilized by treatment with 1% Triton X-100 (v/v, pH 7.4, in PBS) for 5 min. Nonspecific binding was blocked by incubating the samples with BSA (1% w/v in PBS) at 37 °C for 30 min. Actin filaments were stained with FITC-phalloidin (1:200, v/v) for 1 h, followed by nuclear staining with DAPI for 10 min. After washing with PBS, the images were obtained using CLSM.

#### Invasion Assay

In order to observe invasion, spheroids of U87 EGFP and U87 EGFP-HMC3 coculture cells were formed. Initially, the cells were seeded into ultralow attachment plates (5 × 10^3^ cells/100 μL in complete medium) and incubated in a CO_2_ incubator (5% CO_2_, 37 °C). After 72 h, compact spheroids were formed, which were then transferred into hydrogel solution droplets by gentle pipetting. The hydrogel-embedded spheroids were incubated in a CO_2_ incubator for 48 h. Images of the spheroids were taken at 0, 24, and 48 h using CLSM, and the migration distances of cells from the spheroid edges were measured to assess their invasion potential within the hydrogels.

#### Immunofluorescence Staining

After 7 days of culture in the hydrogels, the cells were fixed with paraformaldehyde (4%, w/v) for 15 min at room temperature. The cells were then washed with PBS (10 mM, pH 7.4) and the cell membranes were permeabilized with Triton X-100 (0.1%, v/v in PBST) for 10 min. In order to block nonspecific binding, the cells were incubated with (5% Normal Goat Serum in PBST) for 1h. The cells were washed with goat serum (1%, v/v in PBST) and incubated with primary antibody (rabbit antihuman anti-Ki67 IgG primary antibody, 1:250, v/v in 1% goat serum) overnight at 4 °C. After washing with 1% goat serum, the cells were incubated with secondary antibody (Alexa Fluor 555-conjugated antirabbit IgG secondary antibody, 1:500, v/v in 1% goat serum) and FITC-phalloidin (1:200, v/v) at 37 °C for 1 h.^49^ The cells were washed three times with PBS and counterstained with DAPI for 10 min. The cells were then washed with PBS and examined using CLSM. Fluorescence intensities per cell were determined from CLSM images using ImageJ software.

#### Gene Expression Analysis with RT-PCR

Cells were introduced into hydrogels and cultured for 7 days under two conditions: as GBM monoculture and as GBM–microglia coculture. Total RNA was extracted using the Direct-zol RNA Miniprep Kit (Zymo Research). Hydrogels were homogenized in 1 mL Trizol using sonication (90 s, 10 s on/off), followed by phase separation with 200 μL chloroform and centrifugation (16,000 rpm, 15 min, 4 °C). The aqueous phase was mixed with ethanol and loaded onto spin columns. After DNase I treatment and successive wash steps, RNA was eluted in 50 μL RNase-free water. Concentration and purity were evaluated using a NanoDrop spectrophotometer. cDNA synthesis was performed using the RevertAid First Strand cDNA Synthesis Kit (Thermo Scientific) from 1 μg total RNA in a 20 μL reaction. Quantitative PCR was carried out using PowerUp SYBR Green Master Mix (Applied Biosystems), with specific primers for MMP-2, MMP-9 and GAPDH as a housekeeping gene. Reactions were run under the following conditions: UDG activation at 50 °C for 2 min, initial denaturation at 95 °C for 2 min, followed by 40 cycles of 95 °C for 15 s and 60 °C for 1 min. Melting curve analysis (60–95 °C) was performed to confirm amplification specificity. Gene expression levels were calculated using the 2^–ΔΔCt^ method. Gene-specific primers used for RT-PCR were are listed in Table 1.

**Table 1:** Gene Specific Primer Sequences Used for RT-PCR Analysis

Gene	Sequence
**MMP-2**	Forward: 5′-AGC­GAG­TGG­ATG­CCG­CCTT­TAA-3
	Reverse: 5′-CATT­CCAG­GCAT­CTGC­GATG­AG-3
**MMP-9**	Forward: 5′-GCCACT­ACTG­TGCCT­TTGAG­TC-3
	Reverse: 5′-CCCTC­AGA­GAAT­CGCC­AGTA­CT-3
**GAPDH**	Forward: 5′-GTCTCC­TCTGA­CTTC­AACAG­CG-3
	Reverse: 5′-ACCA­CCC­TGTT­GCTGT­AGCC­AA-3

#### Proteomics Analysis with LS-MS/MS

Proteomic analysis was performed using liquid chromatography-tandem mass spectrometry (LC-MS/MS). Proteomic experiments were outsourced to Acibadem Healthcare Group LABMED (Istanbul, Turkiye). Prior to protein extraction, lyophilized samples were mechanically disrupted using magnetic beads to ensure thorough tissue breakdown. Protein extraction was then carried out using the Universal Protein Extraction Kit (UPX), which contained 4% SDS, 0.1 M DTT, and 0.1 M Tris (pH 7.6). Homogenization was performed in a UPX solution supplemented with a protease inhibitor cocktail. The samples were incubated at 95 °C for 5 min, cooled to room temperature, and stored at 4 °C for 1 h. After centrifugation at 15,000 × g for 10 min, the supernatant was collected and stored at −80 °C. For proteomic profiling, proteins were processed using the filter aided sample preparation (FASP) protocol to remove interfering detergents and enable efficient digestion. Briefly, 50 μg of protein from each sample was loaded onto a 30 kDa molecular weight cut off centrifugal filter unit (Microcon 30, Merck Millipore) and washed with 8 M urea in 0.1 M Tris-HCl buffer (pH 8.5). Proteins were alkylated with 400 mM iodoacetamide (IAA) in the dark at room temperature for 20 min. Excess reagents were removed by repeated centrifugation and washing with urea and then with 50 mM ammonium bicarbonate. Subsequently, the proteins retained on the membrane were digested overnight at 37 °C with MS-grade trypsin (enzyme-to-protein ratio 1:50) in 50 mM ammonium bicarbonate. The resulting peptides were recovered by centrifugation, desalted, and subjected to liquid chromatography–mass spectrometry (LC-MS/MS) analysis. The peptide mixtures were separated using nanoLC and analyzed using a high-resolution mass spectrometer (Xevo G2-XS Qtof, Waters). Raw MS/MS spectra were scanned against the *Homo sapiens* reference proteome from the UniProt database using Progenesis QI for Proteomics (Nonlinear Dynamics). Protein identification was performed with a false discovery rate (FDR) of <1%. Quantitative comparisons were carried out using label-free quantification with normalization based on total ion current. For functional interpretation, Gene Ontology (GO) enrichment analysis was conducted using g:Profiler (https://biit.cs.ut.ee/gprofiler/), incorporating GO Biological Process (BP), Molecular Function (MF), and Cellular Component (CC) terms. Enrichment was assessed, and statistically significant terms were identified with an adjusted p-value threshold of 0.05.

#### Statistics

Data are expressed as mean ± SD. Comparisons between groups were performed using one-way analysis of variance (ANOVA) with Bonferroni’s posthoc test. GraphPad Software (RRID: SCR_002798) was used for statistical analysis. Statistical significance was set at *p* < 0.05.

## 4 Conclusions

This study presents the development of dECM-based hydrogels as an advanced platform for modeling the GBM microenvironment and studying tumor progression. The 1H3D hydrogel successfully mimicked the biochemical composition and mechanical properties of GBM tissue by retaining essential extracellular matrix components and demonstrated superior mechanical performance, such as elastic modulus and compressive modulus, closely resembling GBM tumors. These properties provide an adaptable environment that supports key ECM-tumor interactions, including cell adhesion, proliferation, and invasion. *In vitro* studies revealed that 1H3D supported high cell viability (>90%) over 14 days, with U87 glioblastoma cells exhibiting spindle-like morphologies and interconnected networks, indicative of active ECM and cellular interactions. Additionally, coculture with HMC3 cells further enhanced U87 proliferation and invasion, highlighting the critical role of microglia in driving tumor-stroma dynamics. By replicating key ECM-GBM interactions and enabling the study of GBM-stroma dynamics, the 1H3D hydrogel provides a physiologically relevant platform for 3D tumor modeling. This platform holds promise to investigate GBM progression and evaluate therapeutic strategies that target both tumor cells and the microenvironment.

## Supplementary Material







## Data Availability

Data supporting the findings of this study are available from the corresponding author upon reasonable request.

## Notes

Quantification of sulfated glycosaminoglycans and total protein in brain-derived dECMs; SDS-PAGE profiles of native and decellularized tissues; FTIR spectra confirming lipid removal and protein retention; ^1^H NMR and FTIR spectra of hyaluronic acid and its methacrylated form; images of HAMA/dECM hydrogel before and after UV-induced cross-linking; proteomics analysis of mono- and coculture conditions; includes Figures S1–S6 (PDF)
Proteomic data of dECM Table S1 (XLSX)
Proteomic data of mono and coculture conditions Table S2 (XLSX)
