# Supplementary material for: Development of a Hydrogel Platform with GBM and Microglia: A Potential Glioblastoma Tumor Model
Source: ACS Appl Bio Mater. 2025 Aug 25;8(9):7757–70. doi: 10.1021/acsabm.5c00735 (PMC12442066; doi:10.1021/acsabm.5c00735)
Supplement: Supplementary file 1 [file mt5c00735_si_001.pdf]

Supporting Information

Development of a hydrogel platform with GBM and microglia: A potential glioblastoma tumor model

*Seyma Isik[1,2], Deniz Yucel[2,3,4], Vasif Hasirci[2, 4,5,6]**

[1] Department of Medical Biotechnology, Graduate School of Health Sciences, Acibadem University, Atasehir, 34752 İstanbul, Türkiye

[2] Biomaterials Center, Acibadem University, Atasehir, 34752 İstanbul, Türkiye

[3] Department of Histology and Embryology, Acibadem University, Atasehir, 34752 İstanbul, Türkiye

Departments of [4]Biomaterials and [5]Biomedical Engineering, Acibadem University, Atasehir, 34752 İstanbul, Türkiye

[6] BIOMATEN, Center of Excellence in Biomaterials and Tissue Engineering, Middle East Technical University, Cankaya 06800 Ankara, Türkiye

* Corresponding author: vasif.hasirci@acibadem.edu.tr

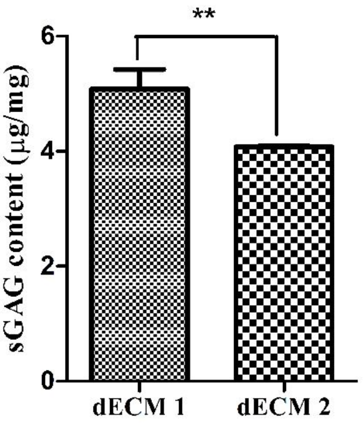

Figure S1. sGAG contents in dECMs. Results are shown as means ± SD of three independent experiments. Statistical analysis was carried out using unpaired t-test. *p < 0.05, **p < 0.01 and ***p < 0.001.

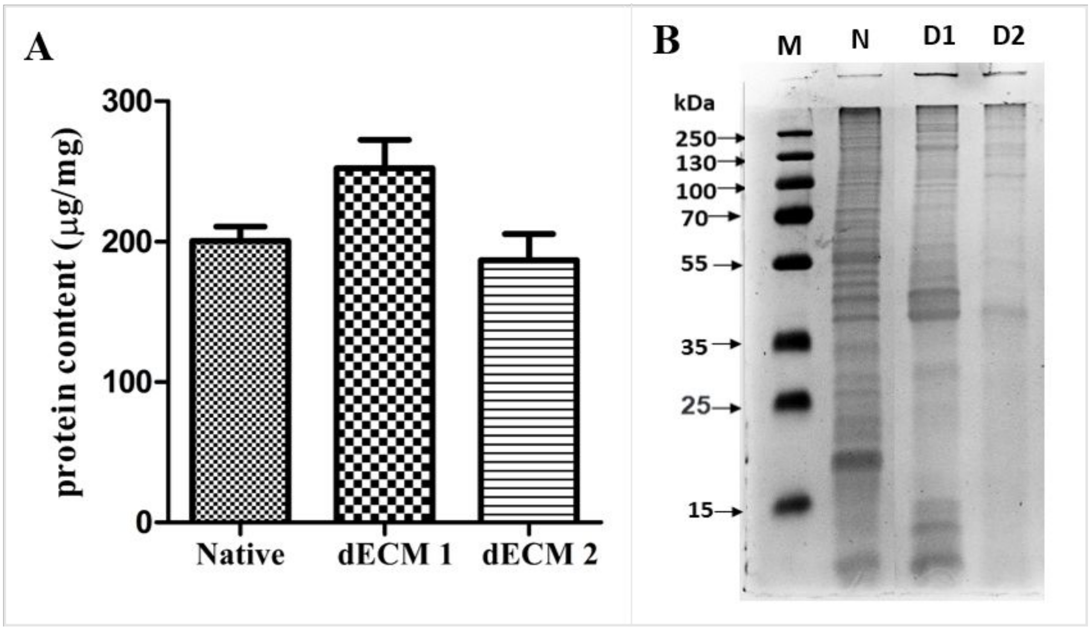

Figure S2. (A) Total protein content of dECMs Results are shown as means ± SD of three independent experiments. (B) SDS-Page pattern of dECMs. Marker, PageRulerTM Plus Prestained Protein Ladder, 10 to 250 kDa; N, Native brain, D1, dECM 1; D2, dECM 2.

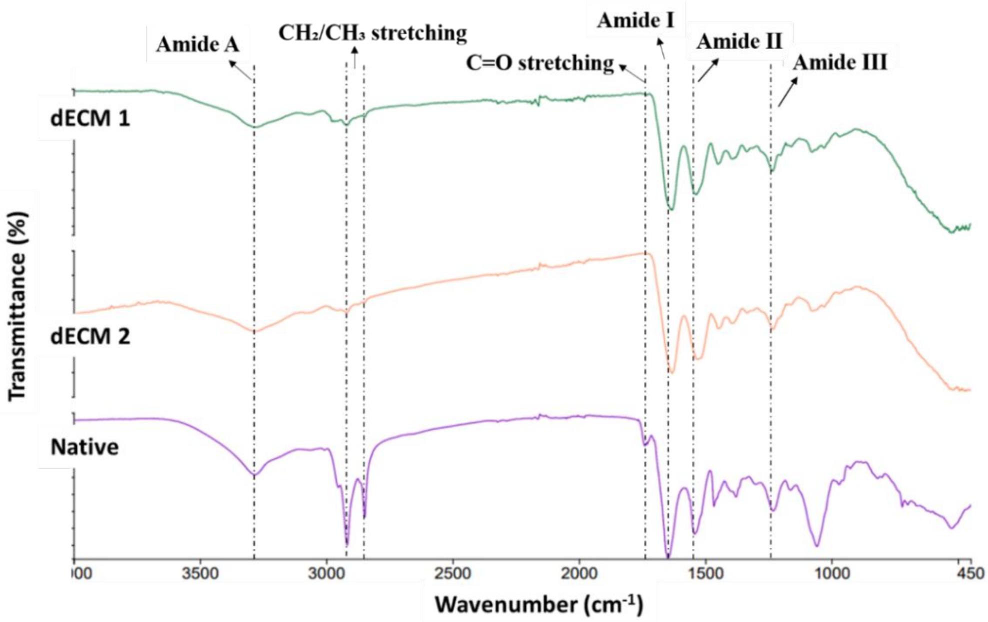

Figure S3. FTIR spectra of the native and decellularized brain tissue. In both the native bovine brain and dECMs, protein-related amide bands were identified. Amide I band, located at 1630 cm^{-1}, Amide II band, at 1525 cm^{-1}, Amide III band, at 1230 cm^{-1} and Amide A band at 3300 cm^{-1} showed the presence of proteins. Lipid related bands, CH_2 and CH_3 stretching vibrations at 2800–3000 cm^{-1} and ester carbonyl (C=O) stretching at 1740 cm^{-1}, were identified in the native brain tissue, in contrast to dECMs, indicating the removal of the lipid components during decellularization.

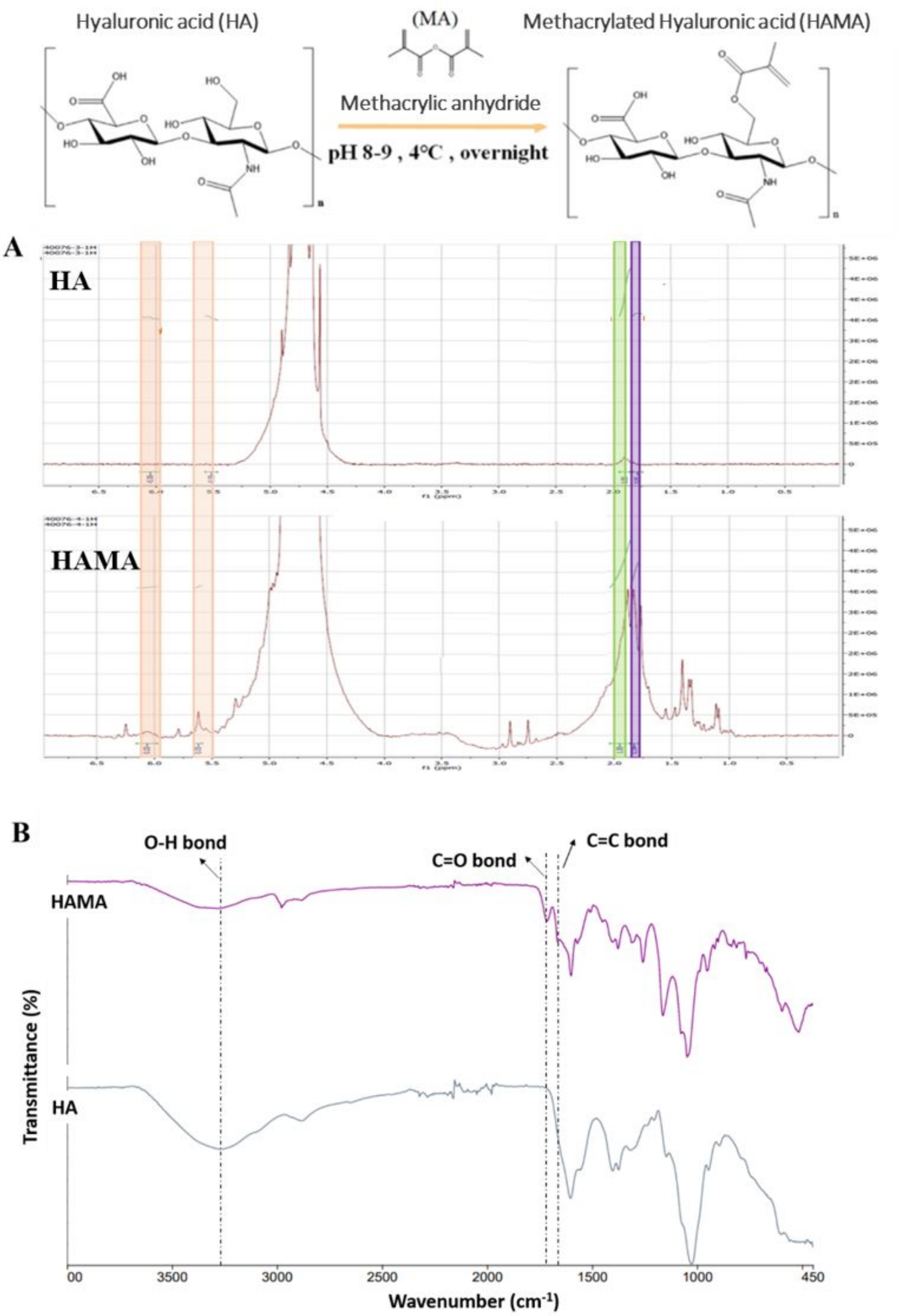

Figure S4. (A) ^{1}H-NMR-spectra of hyaluronic acid and hyaluronic acid methacrylate (two protons of the methylene carbon on the methacrylate group: 6.1 and 5.6 ppm; ten protons of the carbohydrate backbone between: 4.2 to 3 ppm; three protons on the methyl groups of the N-acetyl glucosamine subunit: 1.9 ppm; three protons on the methyl groups of methacrylates:1.85 ppm). (B) FTIR spectra of the hyaluronic acid (HA) and hyaluronic acid methacrylate (HAMA). In contrast to HAMA, methacrylate-related peaks were observed in the HAMA spectra. Peaks at 1720 cm–1 and 1650 cm–1 in HAMA confirmed ester bond formation (C=O stretching) and methacrylation (C=O stretching of methacrylate double bonds). The broad peak at 3200-3600 cm–1 indicates O-H stretching vibrations in hyaluronic acid and HAMA.

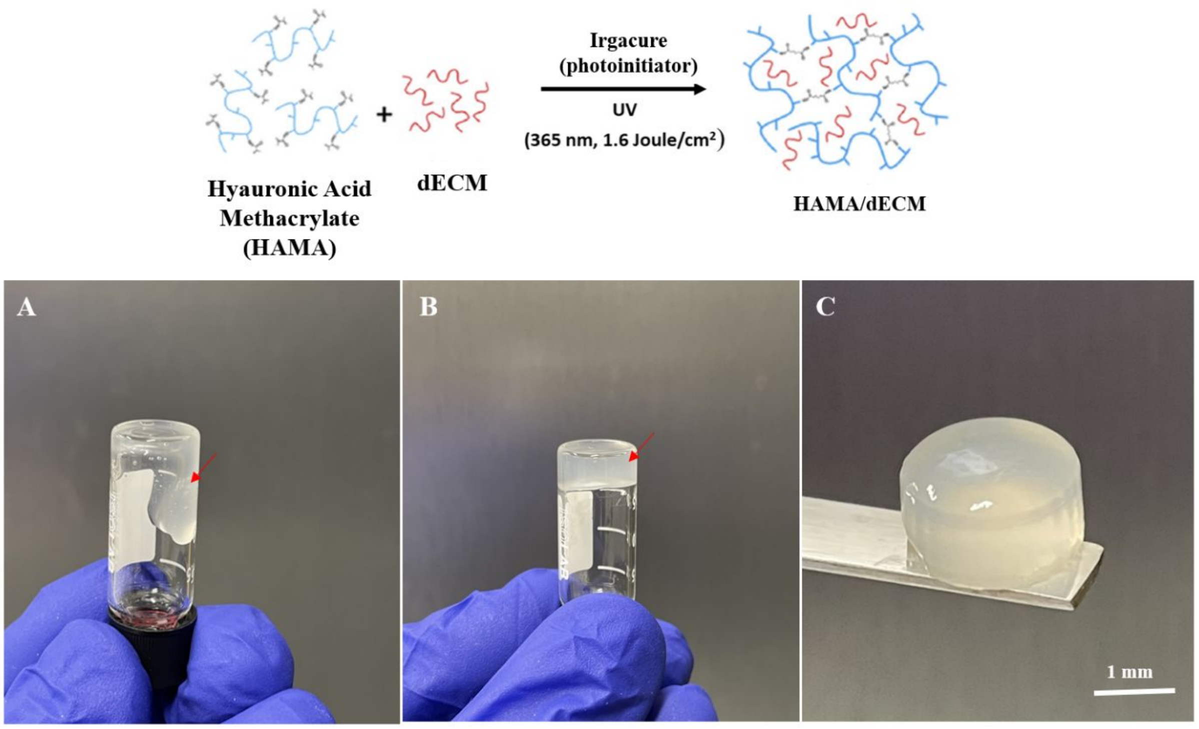

Figure S5. The hydrogel consists of 1% HAMA and 3% dECM was exposed to UV. (A)The hydrogel solution was free flowing before UV exposure and turned into (B) a solid gel after crosslinking with UV, (C) Side view of the hydrogel.

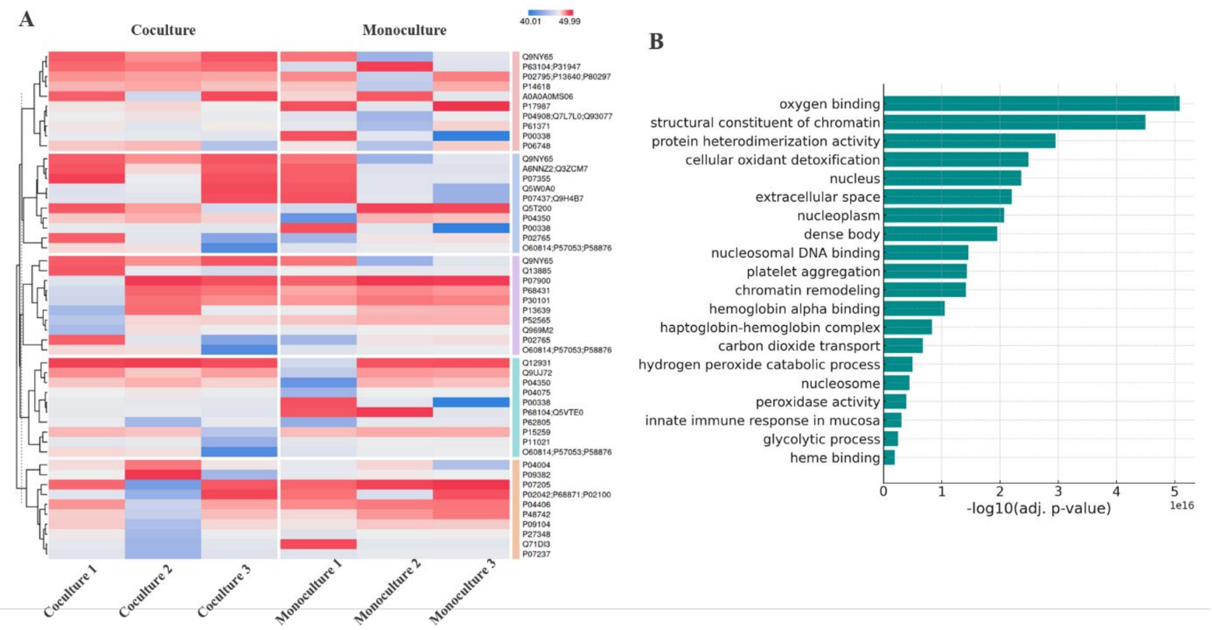

Figure S6. Proteomic comparison of U87 monoculture and U87–HMC3 co-culture. (A) Principal component analysis (PCA) and hierarchical clustering of protein expression profiles obtained from LC-MS/MS-based proteomic analysis. (B) Gene ontology (GO) enrichment analysis of differentially expressed proteins between the co- and monoculture conditions.
